# 超高效液相色谱-串联质谱法定量分析尿液中色氨酸及其代谢产物

**DOI:** 10.3724/SP.J.1123.2020.06022

**Published:** 2021-05-08

**Authors:** Hui LI, Lanchong CUI, Guolei ZHANG, Mengmeng ZHANG, Lili JIAO, Wei WU

**Affiliations:** 1.长春中医药大学, 吉林省人参科学研究院, 吉林 长春 130117; 1. Jilin Ginseng Academy, Changchun University of Chinese Medicine, Changchun 130117, China; 2.长春中医药大学药学院, 吉林 长春 130117; 2. College of Pharmacy, Changchun University of Chinese Medicine, Changchun 130117, China

**Keywords:** 超高效液相色谱-串联质谱, 柱前衍生, 色氨酸-犬尿氨酸代谢途径, 尿液, ultra performance liquid chromatography-tandem mass spectrometry (UPLC-MS/MS), pre-column derivation, tryptophan-kynurenine pathway, urine

## Abstract

基于超高效液相色谱-串联质谱(UPLC-MS/MS)建立定量分析色氨酸(Trp)及代谢产物3-OH-犬尿氨酸(3-OH-Kyn)、3-OH-邻氨基苯甲酸(3-OH-AA)、黄尿酸(XA)、犬尿氨酸(Kyn)、5-羟基吲哚乙酸(5-HIAA)、犬尿喹啉酸(KA)和5-羟色胺(5-HT)的方法,应用该方法分析其在尿样中的含量,探讨排泄规律。将尿样稀释、离心后,加入丹磺酰氯(DNS-Cl)衍生,经Thermo C_18_色谱柱(50 mm×3 mm, 2.7 μm)分离和0.1%甲酸和甲醇梯度洗脱后,采用电喷雾电离(ESI)源,在正离子扫描和多反应监测(MRM)模式下检测。以咖啡酸(CA)为内标,定量分析。结果显示,8种目标化合物的线性关系良好,相关系数(*R* ^2^)≥0.9740,检测灵敏(LOD为0.005~0.5 ng/mL),回收率高(93.24%~107.65%)。采用本方法检测分析了健康志愿者70个尿液样本,在尿样中检测到Trp原型及其7种代谢产物。结果表明,体内的Trp是通过原型和代谢两种方式排泄:Trp原型的含量为5.22~20.88 μg/mL;尿液中经代谢后排泄的Trp量是原型的124%~268%,即体内的Trp主要经代谢后排出体外。方法主要研究了Trp-5-HT和Trp-Kyn两条途径的代谢产物含量,Trp经Kyn降解生成的3-OH-AA和3-OH-Kyn含量较多,即Trp-Kyn是体内Trp的主要代谢途径。方法通过UPLC-MS/MS实现了尿液中Trp及其代谢产物含量的检测,能为临床检查提供技术和理论支持。

## Abstract (en)

Tryptophan (Trp), also known as α-amino *β*-indolepropionic acid, is an essential amino acid, which is involved in various physiological processes. Studies have shown that tumors, infectious diseases, and neurological diseases are accompanied by Trp-related metabolic disorders. Understanding the excretion of Trp and its metabolites in normal individuals is of great significance for treating Trp-related diseases and monitoring the health. A rapid quantitative method was developed based on ultra performance liquid chromatography-tandem mass spectrometry (UPLC-MS/MS). Further, this method was applied to the simultaneous determination of Trp and its metabolites, including kynurenine (Kyn), kynurenic acid (KA), 3-hydroxykynurenine (3-OH-Kyn), 3-hydroxyanthranilic acid (3-OH-AA), xanthurenic acid (XA), 5-hydroxytryptamine (5-HT), and 5-hydroxyindoleacetic acid (5-HIAA). The excretion and amount of target compounds in random urine samples collected from healthy participants were studied using this method. Urine samples were collected from healthy male volunteers (between 20-22 years old) without any diet and exercise restrictions. Urine samples were collected between 11:00-13:00 daily for 10 d. Thereafter, the urine samples were diluted, centrifuged, and subjected to pre-column derivatization with dansyl chloride (DNS-Cl). Caffeic acid (CA) was used as the internal control. Later, the derivatives were detected using triple quadrupole mass spectrometry with electron pray ionization (ESI) in positive and multi reaction monitoring (MRM) modes. The samples were separated using a Thermo C_18_ column (50 mm×3 mm, 2.7 μm) with 0.1% aqueous formic acid aqueous solution and methanol as mobile phases at a flow rate of 0.2 mL/min. The three most abundant ions for each derivative were selected for downstream analysis, and the internal control was used for quantification. The polarity and molecular weight of the compounds were found to be altered effectively after DNS-Cl derivatization treatment. The dansyl group effectively altered the polarities of the derivatives, such that their retention behaviors in the reverse elution system were similar and they were well separated. The interference due to impurities was effectively eliminated using the MRM mode. The results showed significant linear correlation, since the correlation coefficients were greater than 0.9740. The recoveries were between 93.24%-107.65%, and the LODs were 0.005-0.5 ng/mL for the eight compounds. Trp prototype and the seven target metabolites, including 3-OH-Kyn, 3-OH-AA, XA, Kyn, KA, 5-HIAA, and 5-HT generated through Trp-5-HT and Trp-Kyn pathways were detected in the urine samples. These results indicated that Trp was excreted in a prototypic form or after being metabolized. The level of the target compounds in random urine samples of individuals were 0.99-3.72 (3-OH-Kyn), 2.51-21.11 (3-OH-AA), 0.25-1.12 (XA), 0.15-1.53 (Kyn), 0.24-2.58 (KA), 0-0.31 (5-HT), and 2.2-17.94 (5-HIAA) μg/mL. For the same individual, in the state of physical health, the fluctuations of Trp and its metabolites in urine were large. Due to these large fluctuations in the absolute content, the difference between individuals was not significant. The data generated using 70 urine samples revealed that the amount of excreted Trp being metabolized was 124%-268% of prototype, which further indicated that the excretion after metabolism was the major underlying mechanism. Upon comparing the levels of metabolites in the Trp-5-HT and Trp-Kyn pathways, the results indicated that the levels of 3-OH-AA and 3-OH-Kyn generated upon Trp degradation through the Kyn pathway was higher than those of the other products. Trp was degraded via Kyn pathway to produce 3-OH-AA, which was the main metabolite of Trp found to be present in the body. This manuscript detected the levels of Trp and its metabolites, as well as summarized the characteristics of excretion using random urine samples, which could provide valuable information for clinical practice.

色氨酸(Trp)又称*α*-氨基*β*-吲哚丙酸^[1]^,是人体必需的氨基酸之一^[2]^。人体每天需要的Trp来自内源蛋白质的分解和食物的消化吸收,这些Trp除用于蛋白质生物合成外,主要在肝、肾、脑等组织器官发生代谢^[3,4]^。Trp会在吲哚胺-2,3-双加氧酶、单胺氧化酶、犬尿甲酰胺酶等的作用下通过Trp-5-羟色胺(5-HT)和Trp-犬尿氨酸(Kyn)两条主要途径降解并排出体外^[5,6]^。受机体饮食、活动量以及机体状态的影响,每天摄入的Trp以及参与代谢的酶会发生变化,这些细小的变化如果逐渐积累放大,就会造成机体代谢状态的差异,最终导致疾病的发生。研究表明,肿瘤、感染性疾病、神经性疾病等均伴随发生Trp的代谢紊乱^[7,8]^,了解Trp及其代谢产物在正常个体日常状态下的排泄规律对Trp相关疾病的普查、健康状态的监测,以及疾病的早期诊断都具有重要意义。

Trp及其代谢产物在体内含量低,检测难度较大。近年来,利用Trp及其代谢产物的紫外、荧光、电化学性质,可以采用的检测方法有高效液相色谱法(HPLC)^[9]^、高效毛细管电泳法(HPCE)^[10]^和高效液相色谱-质谱法^[11]^(HPLC-MS)等。其中HPLC-MS由于其高度特异性及准确性成为当前的研究热点^[12]^。然而,现有文献关于Trp及其代谢产物的研究报道多集中在疾病个体和正常个体的区分,如Cheng等^[13]^发现肾功能不全患者血浆Trp和其代谢产物的含量较高;Heilman等^[14]^发现血液中Trp及其代谢产物含量可以用于评估帕金森患者病情;吴智明等^[15]^发现大肠癌患者尿液Kyn与Trp比值较对照组显著提高。这些报道研究多采用多时间点收集的尿液进行检测和比较,并不能反映某一时间点机体代谢产物的含量情况。Trp的吸收和排泄是正常机体每天都需要进行的生理过程,受食物摄入和活动量变化的影响较大^[16]^。了解健康志愿者随机尿样中Trp及其代谢产物的水平是Trp等相关代谢产物用于疾病诊断的基础。本实验基于超高效液相色谱-串联质谱法(UPLC-MS/MS),采用丹磺酰氯(DNS-Cl)柱前衍生化技术对目标化合物进行衍生,建立了Trp及其代谢产物的定量分析方法,并应用该方法研究了健康志愿者随机尿样中Trp及其代谢产物的含量,探索其在尿液中的排泄规律,为临床疾病的诊断提供理论支持。

## 1 实验部分

### 1.1 仪器、试剂与材料

Ultimate 3000型超高效液相色谱系统、TSQ Endura三重四极杆质谱仪(Thermo Fisher Scientific公司,美国); AG 22331低温高速离心机(Eppendorf公司,德国); MTN-2800D氮吹浓缩装置(天津奥特赛斯仪器有限公司,中国); SQP电子分析天平(北京赛多利斯科学仪器有限公司,中国); RCT-3200超纯化水机(莱博帕特科技发展有限公司,中国)。

3-OH-犬尿氨酸(3-OH-Kyn,纯度98%)(美国Sigma公司)、Trp(纯度>98.0%)、5-HT(纯度>98.0%); 3-OH-邻氨基苯甲酸(3-OH-AA,纯度98%,上海麦克林生化科技有限公司);黄尿酸(XA,纯度>96.0%,梯希爱上海化成工业发展有限公司);犬尿喹啉酸(KA,纯度>98.0%,上海源叶生物科技有限公司); Kyn(纯度>98.0%,阿拉丁试剂(上海)有限公司); 5-羟吲哚乙酸(5-HIAA,纯度>98.0%,美国Alfa Aesar公司);肌酐(Cr,纯度>98.0%,上海源叶生物科技有限公司);内标咖啡酸(CA,纯度99.9%,日本TCI公司); DNS-Cl纯度99%,北京百灵威科技有限公司);甲醇、甲酸、乙腈(色谱纯,美国Tedia公司)。

尿液样本的采集对象为吉林省长春市的7名健康男性,年龄为20~22岁,所有参与志愿者在采样前均详细阅读并签署了知情同意书。

### 1.2 实验方法

1.2.1 色谱及质谱条件

色谱柱:Thermo C_18_色谱柱(50 mm×3 mm, 2.7 μm);柱温:35 ℃;样品室温度:4 ℃;流动相:A为0.1%甲酸水溶液,B为甲醇;流速:0.2 mL/min。梯度洗脱程序:0~3.0 min, 30%B; 3.0~9.0 min, 30%B~95%B; 9.0~12.0 min, 95%B; 12.0~12.5 min, 95%B~30%B; 12.5~17.0 min, 30%B。进样量:2 μL。

采用电喷雾电离方式进行离子化,正离子、多反应监测(MRM)模式扫描。扫描范围:*m/z* 100~1000;喷雾电压:4000 V;鞘气压力:6.125 MPa;辅助气压力:0.875 MPa;传输毛细管温度:350 ℃;雾化器温度:300 ℃;针泵进样。数据采用Thermo Xcalibar软件进行分析。待测物的碰撞能量(CE)、RF透镜电压(RF lens)和其他质谱参数见表1。

**表 1:** 目标化合物的质谱参数

Compound	*t*_R_/min	*M*_r_	Number of dansyl group	Precursor ion (*m/z*)	Product ion (*m/z*)	Collision energy/eV	RF lens
3-OH-Kyn	3.86	224.08	2	691.14	171.14^*^	37.54	98.31
					456.15	27.48	
					349.37	14.98	
3-OH-AA	4.25	153.04	1	387.09	171.15^*^	17.59	85.73
					216.19	15.36	
					341.50	15.21	
XA	4.78	205.04	1	439.06	170.25^*^	19.81	154.31
					268.16	9.89	
					393.14	12.37	
Kyn	5.30	208.08	1	442.12	170.15^*^	22.21	80.25
					251.14	11.32	
					271.04	9.22	
Trp	5.38	203.80	1	438.18	170.89^*^	23.33	122.21
					200.02	17.63	
					283.56	16.51	
5-HIAA	6.22	191.06	1	425.11	171.15^*^	22.39	240.83
					205.89	19.33	
					380.35	12.54	
KA	6.51	189.04	1	423.51	170.44^*^	18.72	120.39
					252.10	11.65	
					378.22	12.25	
5-HT	7.75	179.06	2	643.34	171.13^*^	37.95	88.75
					380.10	15.66	
					393.02	28.77	
CA (IS)	7.78	182.06	2	647.09	171.11^*^	27.39	145.28
					234.57	20.72	
					413.07	18.70	

RF: radio frequency; * Quantitative ion

1.2.2 样品的收集与处理

于每天11∶00~13∶00在洁净容器中收集中段尿液10 mL,取1 mL,加入100 μL 1%甲酸水溶液,混匀后,以5000 r/min离心10 min,取上清200 μL,于-80 ℃避光冷冻,备用,分析时,尿样于4 ℃下解冻。向尿液样品中加入50 μL 100 μg/mL的CA和含8 mg/L DNS-Cl的乙腈溶液200 μL,混匀,加入400 μL 0.1 mol/L Na_2_CO_3_-NaHCO_3_缓冲溶液,涡旋,于60 ℃金属浴加热20 min。再次向反应体系中加入16 μL 0.5 mol/L三乙胺溶液。反应30 min后加入15%甲酸水溶液16 μL,终止反应,过0.22 μm微孔滤膜,备用。衍生反应的结构通式见图1。

**图 1:**
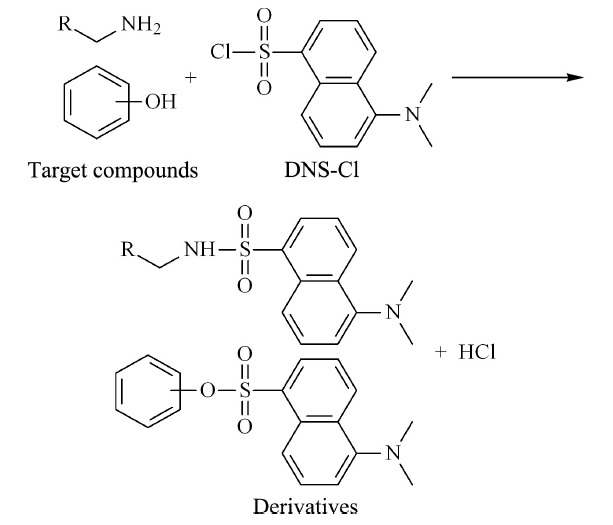
丹磺酰氯的衍生反应

配制含有3.0 mmol/L MgCl_2_·6H_2_O、3.8 mmol/L CaCl_2_·2H_2_O、14.5 mmol/L Na_2_SO_4_、72.1 mmol/L NaCl、2.2 mmol/L C_6_H_5_Na_3_O_7_·2H_2_O、18.7 mmol/L KH_2_PO_4_、0.15 mmol/L Na_2_C_2_O_4_、19.3 mmol/L KCl、17.2 mmol/L NH_4_Cl、41.6 mmol/L 尿素和9.3 mmol/L Cr的人工尿样(pH=7.4)模拟空白基质。在空白基质中加入不同浓度的标准溶液作为质控(QC)样本用于监控分析序列的重复性和稳定性,处理方法同上。

## 2 结果与讨论

### 2.1 色谱及质谱条件的优化

本实验所测定的化合物含有氮原子,相对分子质量较小,在正离子扫描模式下,[M+H]^+^强度很弱,难以检测,且易发生源内裂解,检测难度较大。同时,由于极性原因,目标化合物与尿液中强极性化合物一同流出,基质效应较大。本实验以丹磺酰氯为衍生化试剂,采用柱前衍生的方法对尿液中Trp及其代谢产物进行衍生。丹磺酰氯是一种强荧光剂,基团上的磺酰基能够与伯胺、仲胺以及酚羟基发生取代反应,提高化合物的相对分子质量,同时丹磺酰氯衍生后还可以改善待测物的极性,延长保留时间,提高检测灵敏度。待测物的二级质谱图见图2。

**图 2:**
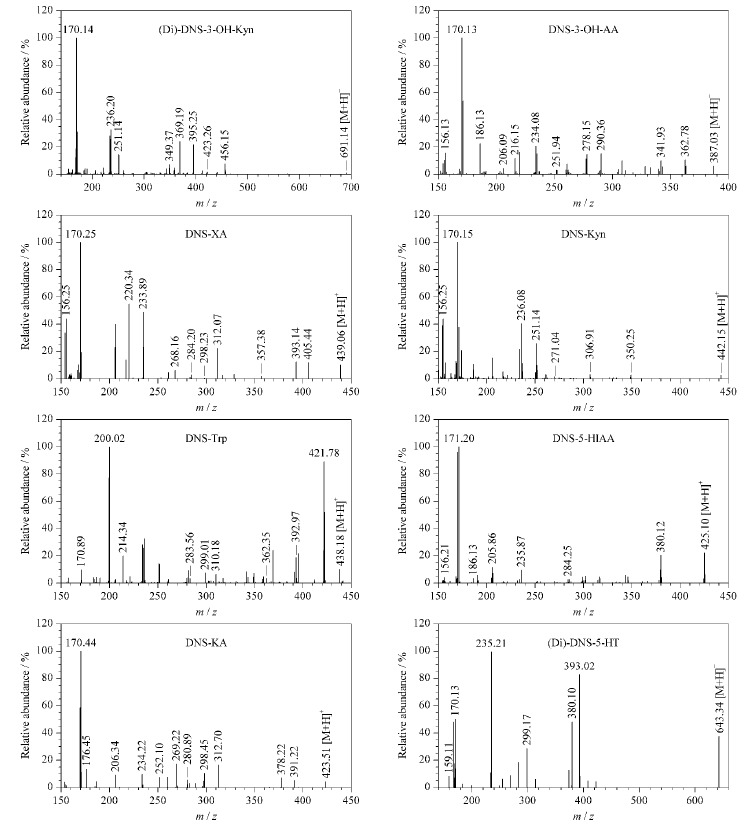
衍生产物的二级质谱图

### 2.2 方法学考察

待测化合物属于内源性物质,本研究依据《中国药典》(2015版)中“生物样品定量分析方法验证指导原则”,采用人工尿样作为空白基质,向其中添加标准品的方法进行方法学验证,考察了方法的专属性、线性范围、精密度、稳定性和回收率。

2.2.1 专属性

精密量取人工尿液样本,加入不同浓度的混合标准溶液,建立空白基质匹配标准曲线。按1.2.2节处理空白人工尿液和实际尿液,并将混合标准溶液加入人工尿液中,分别得到空白人工尿液、实际尿液和加标尿样的提取离子流色谱图(见图3)。

**图 3:**
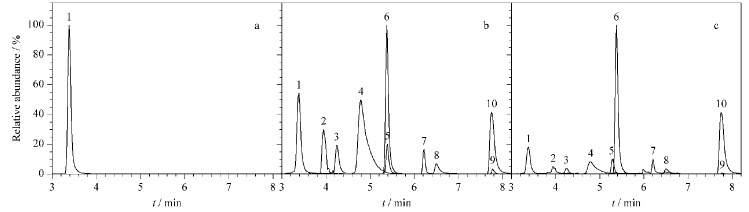
衍生产物的提取离子流色谱图

通过提取离子流色谱图分析可知,人工尿液中只有Cr峰,而无其他目标化合物(见图3a),图3b中待分析物的保留时间与图3c中标准品一致。说明本方法的专属性良好。

2.2.2 线性范围与检出限

在优化后的条件下对系列混合标准溶液进行分析,并以浓度为横坐标、目标化合物与内标(CA)峰面积比值为纵坐标,绘制标准曲线。结果表明,8种目标化合物各自范围内有良好的线性关系,相关系数(*R*^2^)≥0.9740。检出限(LOD)以3倍的信噪比计算,8种目标化合物的LOD为0.005~0.5 ng/mL(见表2)。

**表 2:** 目标化合物的线性方程、相关系数、线性范围、检出限、回收率和精密度

Compound	Linear equation	*R* ^2^	Linear range/ (μg/mL)		LOD/ (ng/mL)	Spiked content/ (μg/mL)	Recovery/%	RSDs (*n*=6)/%		
								Intra-day		Inter-day
3-OH-Kyn	*Y*=0.0082*X*-0.046	0.9764	0.1-	5.0	0.01	0.1	105.41	1.31		0.61
						2.5	106.22	2.54		1.23
						5	98.47	3.11		3.26
3-OH-AA	*Y*=0.0009*X*-0.0310	0.9873	0.5-	25	0.1	0.5	93.24	0.78		0.78
						10	106.24	3.11		3.11
						20	102.61	2.21		2.21
XA	*Y*=0.0645*X* -0.0683	0.9740	0.1-	5.0	0.05	0.1	102.92	0.51		0.51
						2.5	104.23	2.14		2.14
						5	96.97	1.16		1.16
Kyn	*Y*=2.9064*X*-0.1972	0.9941	0.5-	25	0.1	1	95.96	1.23		1.23
						12	105.44	2.11		2.11
						25	103.12	1.11		1.11
Trp	*Y*=0.5550*X*+0.1972	0.9954	1-	50	0.5	2	107.65	2.14		3.59
						20	105.94	3.12		3.12
						40	102.21	1.45		1.45
5-HIAA	*Y*=4.5184*X*+0.8491	0.9797	0.2-	10	0.01	0.2	96.77	0.85		0.85
						4	104.33	2.33		2.33
						8	107.25	2.54		2.54
KA	*Y*=0.0677*X*-0.0153	0.9908	0.1-	5.0	0.05	0.1	96.77	0.75		0.75
						2	98.99	2.69		2.69
						4	102.21	1.78		1.78
5-HT	*Y*=0.0105*X*+0.0457	0.9998	0.01-	0.5	0.005	0.02	97.47	0.99		0.99
						0.25	104.43	1.23		1.23
						0.5	103.32	1.25		1.25

*Y*: peak area ratio of analyte to internal standard; *X*: mass concentration, μg/mL.

2.2.3 回收率和精密度

在空白基质(人工尿样)中分别添加高、中、低3种不同水平的混合标准溶液,进行方法回收率的验证,各目标化合物加标水平见表2。结果表明,目标化合物的回收率为93.24%~107.65%。每种水平各6份,连续测定3 d,计算日内和日间精密度,结果分别为0.51%~3.12%和0.51%~3.59%。

2.2.4 稳定性

取人工尿液样本,加入线性范围中高点和低点两个水平的混合标准溶液,每个水平制备3个平行样本,分别考察样品在室温放置6 h、反复冻融3次、-80 ℃冻存10 d及经处理后室温放置24 h后的稳定性。

结果表明,在上述考察条件下,目标化合物含量的标准偏差均小于15%,表明样品在上述条件下均保持稳定。

### 2.3 尿液测定结果

将尿液进行处理和分析,得到尿液中目标化合物的含量。7名健康志愿者的尿样连续测定10 d,结果见图4。

**图 4:**
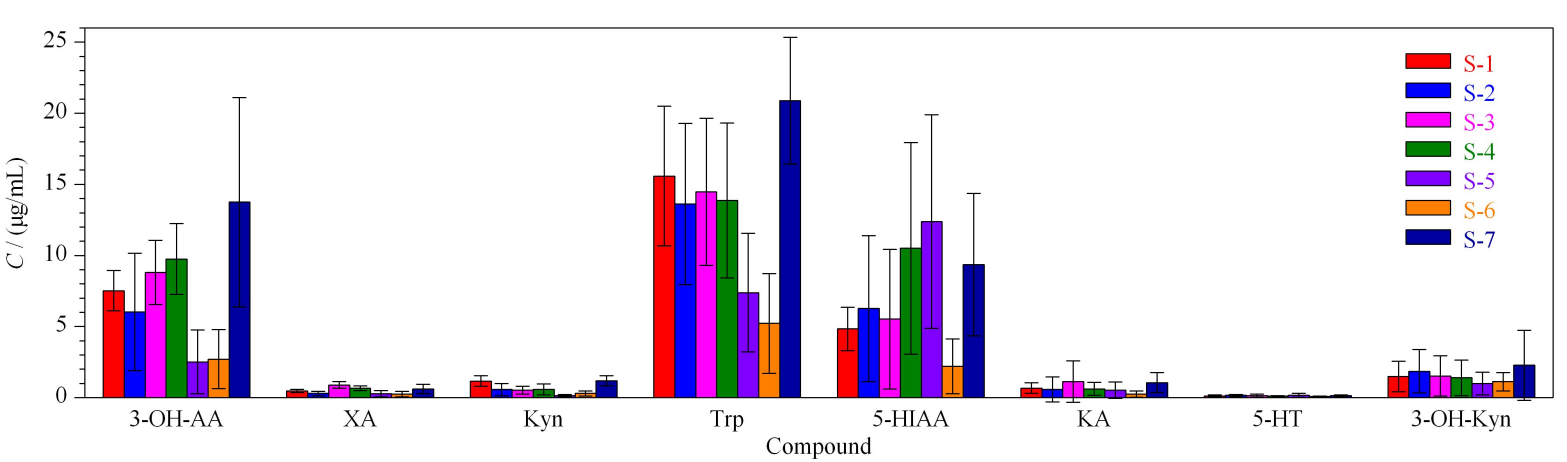
7名志愿者尿样中目标化合物的含量(平均值±SD,*n*=10)

尿液中的Trp主要来源于食物中外源性的氨基酸和机体内源性蛋白质的分解^[17]^,由于志愿者未受运动和饮食限制,尿样的色氨酸含量可能会受到饮食和运动影响。

经测定,志愿者的尿中Trp的含量波动范围为1.71~25.34 μg/mL,经代谢后排泄的Trp是原型量的124%~268%。在Trp的两条代谢途径中,经Kyn途径生成Kyn、KA、3-OH-Kyn、3-OH-AA和XA,尿液中的含量分别是0.99~3.72 (3-OH-Kyn)、2.51~21.11 (3-OH-AA)、0.25~1.12 (XA)、0.15~1.53 (Kyn)和0.24~2.58 (KA) μg/mL;经5-HT途径生成的5-HT和5-HIAA,含量分别为0~0.31 μg/mL(5-HT)和2.2~17.94 μg/mL(5-HIAA)。Kyn途径代谢产物含量是5-HT代谢途径相关产物的104%~176%,说明Trp经Kyn降解生成3-OH-AA和3-OH-Kyn的是Trp的主要代谢产物。尿中的Trp-Kyn代谢产物的测定结果与已有报道^[18]^结果一致。经统计学分析,受试者6(S-6)和受试者7(S-7)间差异较明显(*P*<0.05),其余受试者均不存在显著性差异。

有文献报道^[19,20,21]^将肌酐作为内参,通过其他化合物浓度与肌酐含量的比值来进行计算。本方法也以肌酐含量为基准,比较了目标化合物含量在不同尿样中的差异,并没有发现不同个体尿样中目标化合物含量有显著性差异。

## 3 结论

本文基于UPLC-MS/MS技术建立了一种测定Trp及其代谢产物的定量方法。该方法采用DNS-Cl对目标化合物进行衍生,方法灵敏度高,重复性好,可以实现尿液中目标化合物的准确定量。
